# Optoelectronic Properties of α-MoO_3_ Tuned by H Dopant in Different Concentration

**DOI:** 10.3390/ma15093378

**Published:** 2022-05-08

**Authors:** Xi Huang, Xin Xu, Jiawei Huang, Zheyu Zhang, Yujia Gao, Zhengli Lu, Zhenyuan Wu, Tian Luo, Yating Cai, Yating Qu, Pengyi Liu, Cuiying Hu, Tingting Shi, Weiguang Xie

**Affiliations:** Siyuan Laboratory, Guangzhou Key Laboratory of Vacuum Coating Technologies and New Energy Materials, Guangdong Provincial Engineering Technology Research Center of Vacuum Coating Technologies and New Energy Materials, Department of Physics, Jinan University, Guangzhou 510632, China; huangxi1@stu2019.jnu.edu.cn (X.H.); xuxin@stu2021.jnu.edu.cn (X.X.); hjwttt@stu2019.jnu.edu.cn (J.H.); zheyuzhang2001@163.com (Z.Z.); m13750070026@163.com (Y.G.); zhenglilu@stu2020.jnu.edu.cn (Z.L.); wuzhenyuan@stu2020.jnu.edu.cn (Z.W.); luotians@outlook.com (T.L.); cyt18023211948@163.com (Y.C.); yatingqu1108@163.com (Y.Q.); tlpy@jnu.edu.cn (P.L.)

**Keywords:** α-MoO_3_, Insertion, H doping modulation, layer structure, optoelectronic properties

## Abstract

The optoelectronic properties of layered α-MoO_3_ are greatly limited due to its wide band gap and low carrier concentration. The insertion of hydrogen (H) can effectively tune the band structure and carrier concentration of MoO_3_. Herein, first-principles calculations were performed to unravel the physical mechanism of a H-doped α-MoO_3_ system. We found that the modulation of the electronic structure of H-doped MoO_3_ depends on the doping concentration and position of the H atoms. It was found that the band gap decreases at 8% doping concentration due to the strong coupling between Mo-4d and O-2p orbits when H atoms are inserted into the interlayer. More interestingly, the band gap decreases to an extreme due to the Mo-4d orbit when all the H atoms are inserted into the intralayer only, which has a remarkable effect on light absorption. Our research provides a comprehensive theoretical discussion on the mechanism of H-doped α-MoO_3_ from the doping positions and doping concentrations, and offers useful strategies on doping modulation of the photoelectric properties of layered transition metal oxides.

## 1. Introduction

Two-dimensional (2D) nanomaterials, applied as optoelectronic devices or energy storage elements, are expected to play an important role in nanoelectronic devices [1,2,3]. In the past few decades, a large number of two-dimensional materials such as graphene, molybdenum disulfide, and phosphorus-based materials have been found by researchers. These materials are considered as promising candidates for future electronics. Graphene has very high electron mobility [4], but zero band gap limits its practical application in transistor devices [5]. Typical transition metal dihalide compounds (TMDCs), such as MoS_2_, can achieve ultra-high switching ratios [6,7,8], but the device has low carrier mobility and weak light absorption. Additionally, phosphorus-based two-dimensional materials with high mobility were also found, but they decompose rapidly in a realistic environment [9,10]. Layered transition metal oxide (TMO) materials attracted the attention of researchers due to their excellent stability and rich physical and chemical properties [11]. As one of the important members of TMO, cubic molybdenum trioxide (α-MoO_3_) has attracted extensive attention because of its extraordinary environmental stability and tunable photoelectric characteristics, which have been widely used in energy storage [12], solar cells [13], sensors [14], photoelectric devices [15], and other fields.

As we know, two-dimensional α-MoO_3_ crystal has a unique layered structure and a wide band gap (2.8–3.0 eV), resulting in low intrinsic carrier concentration and low conductivity and making it difficult to be directly applied to electronic devices [16,17,18]. Therefore, the main challenge of developing nano α-MoO_3_ functional devices is to effectively adjust the band gap and carrier concentration of α-MoO_3_. Doping is an effective method to improve its photoelectric properties [19,20,21]. Previous studies have shown that ion insertion (usually hydrogen and alkali metal ions) can control the electron band gap of α-MoO_3_, and one of the most effective post-treatments for improving the band gap of the pristine α-MoO_3_ is hydrogenation [11,20,22]. Kourosh, Kalantar-zadeh, and others regulate their energy band structure by doping molybdenum oxide with hydrogen ions. They believe that most of the inserted H^+^ combines with edge-shared oxygen and terminal oxygen to form OH_2_ groups and convert MoO_3_ to H_x_MoO_3_. However, the OH_2_ group is not stable in the presence of environmental disturbances (such as heat) and is finally released from the original position in the lattice, leaving oxygen vacancies followed by the formation of nonstoichiometric MoO_3−x_ [23]. Furthermore, Maria Vasilopoulou et al. used Coulomb charge to reduce the band gap of two-dimensional MoO_3_. These charges can be introduced either by inserting H^+^ to form H_x_MoO_3_ or by reducing MoO_3_ to MoO_3−x_. Finally, α-MoO_3_ obtained higher carrier mobility at about 1100 cm^2^V^−1^s^−1^ while maintaining the original high work function [24]. In addition, hydrogen doping can significantly improve the conductivity of α-MoO_3_ and its optical response in the visible region [18]. As an interface layer, α-MoO_3_ can also greatly improve the performance of organic optoelectronic devices [25]. However, these studies have not fully discussed the physical mechanism of hydrogen ion insertion in MoO_3_ and the chemical bonding with three different coordinate oxygen in doped molybdenum oxide. Few theoretical studies have reported the effects of different combinations of H atoms and three kinds of oxygen atoms on the band structures, and discussed the doping position and concentration of hydrogen ion on tuning the photoelectric properties of α-MoO_3_. 

H-doped α-MoO_3_ is the effective way to tune the band structure and photoelectric properties [24,25,26]. It is well known that band gap modulation depends on the type and doping concentration of impurity atoms [27,28]. Herein, we theoretically established a series of doped models and focused on the physical regulation mechanisms under different bonding modes. By systematically studying the internal mechanism of α-MoO_3_ after doping H atoms and the corresponding changed band structures, we found that the doping position associated with the concentration of impurity plays an important role in the photoelectric properties of α-MoO_3_. This paper not only reveals the internal physical mechanism of H doping, but also provides useful theoretical guidance for the actual H doping of α-MoO_3_.

## 2. Methods

All first-principles calculations were performed using the Vienna ab initio simulation package (VASP version 5.4.4) based on density functional theory (DFT). Considering the strong magnetism of Mo, GGA + U (generalized gradient approximation method and modified by the Hubbard model) is used in the calculation of electronic structure [29]. The pseudo-potential was considered in calculations through the projector-augmented wave (PAW) method. The cut-off energy of plane waves was set at 400 eV. The Brillouin zone integration was performed using a 2 × 3 × 4 k-point mesh. In order to make the doping concentration closer to the actual situation, we established a 2 × 3 × 1 α-MoO_3_ supercell to calculate the doping of a single H atom.

## 3. Results and Discussion

**Figure 1:**
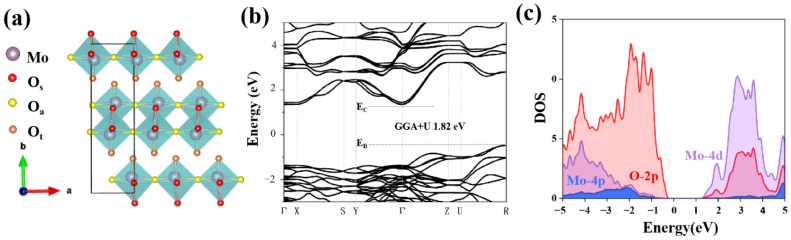
(**a**) The crystal structure of two-dimensional α-MoO_3_. (**b**) The calculated band structures of α-MoO_3_ unit cell. (**c**) The partial density of states (PDOS) of intrinsic α-MoO_3_.

α-MoO_3_ is a two-dimensional layered material with a unique orthogonal structure (space group: Pbnm, a = 3.697 Å, B = 13.864 Å and C = 3.963 Å). It has a distorted octahedral structure, and each layer is connected by van der Waals interaction. It also has three different types of oxygen, namely asymmetric oxygen, symmetric oxygen and terminal oxygen, which are labeled as O_a_, O_s_ and O_t_, respectively. Firstly, the electronic structure of the unit cell is calculated, the band structure shows an indirect band gap, and the maximum of valence band and the minimum of conduction band are at high symmetric K-points R and Γ, respectively. The band gap value corrected by the GGA + U method was 1.82 eV, which is basically consistent with the previous literature, and the actual band gap value is usually underestimated [30,31,32]. In addition, as shown in Figure 1c, it can be seen from the density of states that the valence band of molybdenum trioxide is mainly contributed by O-2p orbital electrons, while the conduction band is mainly contributed by Mo-4d orbital electrons. This analysis is also consistent with the previous studies of α-MoO_3_ [27].

**Figure 2:**
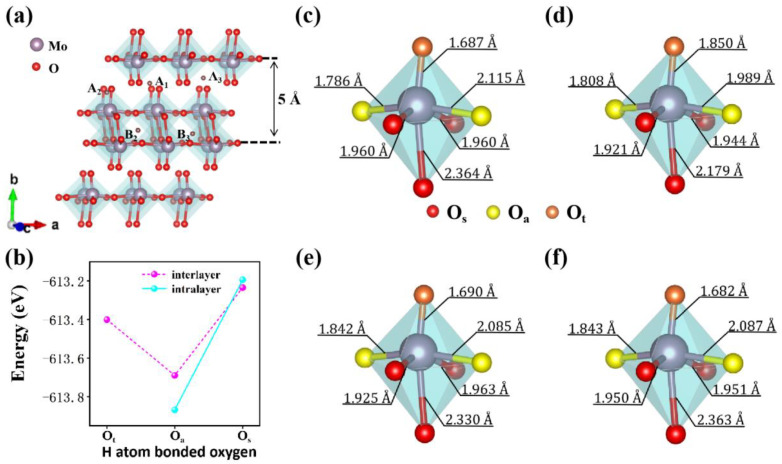
(**a**) Schematic diagram of doping sites. (**b**) System energy at different doping sites. (**c**) Intrinsic α-MoO_3._ (**d**) The H atom bonded to O_t_ at position A_1_. (**e**) The H atom bonded to O_a_ at position A_3_. (**f**) The H atom bonded to O_a_ at position B_2_.

As shown in Figure 2a, to find the most stable doping position, five sites were selected in interlayer or intralayer according to the symmetry of α-MoO_3_. For the interlayer positions, H atoms bonded with O_t_, O_s_ and O_a_ when they doped into A_1_, A_2_ and A_3_ sites, respectively. For the intralayer positions, H atoms bonded with O_a_ and O_s_ when they doped into B_2_ and B_3_ sites, respectively. As we can see in Figure 2b, in comparison with the total energies of the different systems it was found that when H is doped at the concentration of 4%, it is easier to form a coordination bond with O_a_, and the intralayer doped system is more stable than the interlayer doped system because the corresponding system energy is relatively lower (see Table S1 for detailed data). This is consistent with the study by Ritter et al., which indicated that H atoms first fill the intralayer positions on the asymmetric oxygen atoms, and then start to fill the terminal oxygen sites when the H atom concentration x > 0.85 (H_x_MoO_3_) [33]. In addition, by comparing the structure of molybdenum trioxide octahedron before and after H doping (as shown in Figure 2c–f), we found that MoO_6_ octahedron produced varying degrees of distortion, which can be clearly seen in the change of O_a_-Mo-O_a_ bond length. Among them, due to the weak van der Waals force between layers, the strong coordination bond between H and O_t_ breaks the original central symmetry of MoO_6_ octahedron. Compared with a non-doping state, the longest Mo-O_a_ bond length is shortened by 0.126 Å and the shortest Mo-O_a_ bond length is increased by 0.022 Å. (Figure 2d) The bonding of H atom and O_a_ in interlayers or intralayers not only leads to small octahedral distortion, but also has the effect of making the octahedral structure more symmetrical. In conclusion, we can confirm that the most stable doping site at low concentration H is B_2_, where H forms a coordination bond with O_a_ in the intralayer. Therefore, our research mainly focused on the most stable and metastable sites B_2_ and A_3._ In addition, because the H ion radius (0.25 Å) is very small, its insertion does not cause significant lattice distortion, which remains the original two-dimensional structure of α-MoO_3_.

**Figure 3:**
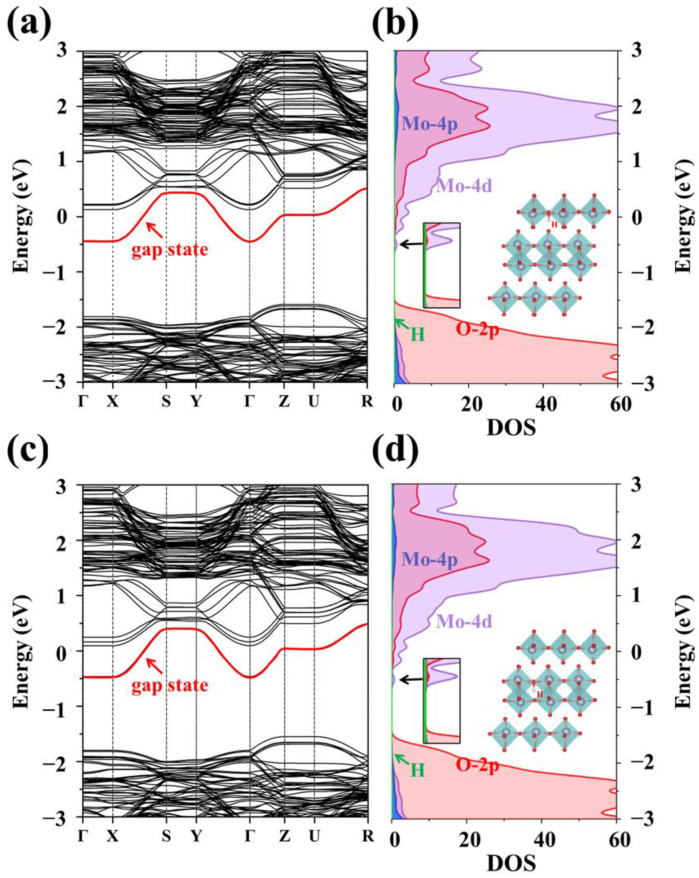
(**a**) Band structure of interlayer doped H atoms. (**b**) Band structure of intralayer doped H atoms. (**c**) Density of states of interlayer doped H atoms. (**d**) Density of states of intralayer doped H atoms.

H doping not only leads to small deformation of α-MoO_3_, but also plays an important role in modulating the energy band structure of α-MoO_3_. Figure 3a shows the band structure of the system when the H atom is doped at A_3_ position. Compared with the band structure of intrinsic α-MoO_3_, H insertion introduces an obvious impurity level near the conduction band, which provides more transition levels for carrier transition in the wide gap band. From the position of relative Fermi level, it was found that doping makes the minimum of conduction band and the maximum of valence band move downwards, which shows the behavior of n-type doping. The density of states in Figure 3b also confirms the existence of gap states which are near the bottom of the conduction band, composed of Mo-4d orbit and weak O-2p orbit coupling. In addition, when impurity H is doped at the most stable B_2_ position, it can be clearly seen in the energy band and density of states that similar impurity levels are generated near the bottom of the conduction band, and are mainly contributed by Mo-4d orbital electrons (Figure 3c,d). From the calculated results, low concentrations of H doping can tune the band gap through shallow level defects, but the modulation effect is weak. Therefore, we tried to increase the H doping concentration and observe the different optoelectronic influence on band gap.

**Figure 4:**
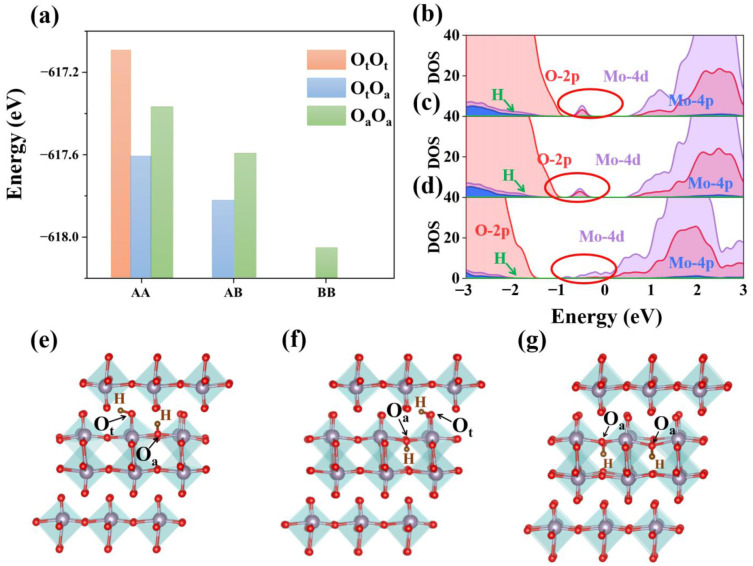
(**a**) Energy diagram of three systems. (**b**) Density of states of AA site. (**c**) Density of states of AB site. (**d**) Density of states of BB site. (**e**) AA site bonding structure. (**f**) AB site bonding structure. (**g**) BB site bonding structure.

Firstly, we calculated the most stable doping position of H at 8% concentration. Because α-MoO_3_ has two different doped positions, interlayer and intralayer, we examined three doping styles: H atoms that are both doped in interlayer as AA site, H atoms that are both doped in intralayer as BB, and H atoms that exist both interlayer and intralayer as AB. As shown in Figure 4a, the total energy of the doped system showed that the most stable doping situation was BB site (see Table S2 for systems energy of each doping site). By further analyzing the bonding characteristics, we found that under AA or AB doping, H tends to bond alternately with O_t_ and O_a_ in the same MoO_6_ octahedron, which is quite different from the situation under low concentration doping. In the most stable BB doping mode, H retains the bonding mode at low concentration and tends to coordinate with O_a_. In addition, by measuring the bond length between O_a_ and adjacent Mo at AB site, we found that this doping method greatly improved the symmetry of the O-Mo bond formed between asymmetric oxygen and Mo. The Mo-O_a_ bond length in MoO_6_ octahedron changed from 1.79 Å to 2.05 Å and 2.11 Å to 2.09 Å, respectively. The effect of H insertion on the interlayer spacing was also investigated. Interlayer spacing of α-MoO_3_ is 5.000 Å (Figure 2a). When doping concentration is 4% or 8%, the interlayer spacing of α-MoO_3_ increased to 5.012 Å and 5.014 Å, respectively, which indicates that insertion of H atoms results in only minor interlayer spacing increases due to its small ionic radius. In order to explore the reasons for the formation of different doping modes under a high concentration of H atoms, we calculated their electronic structures to unravel the different modulation of band gaps.

**Figure 5:**
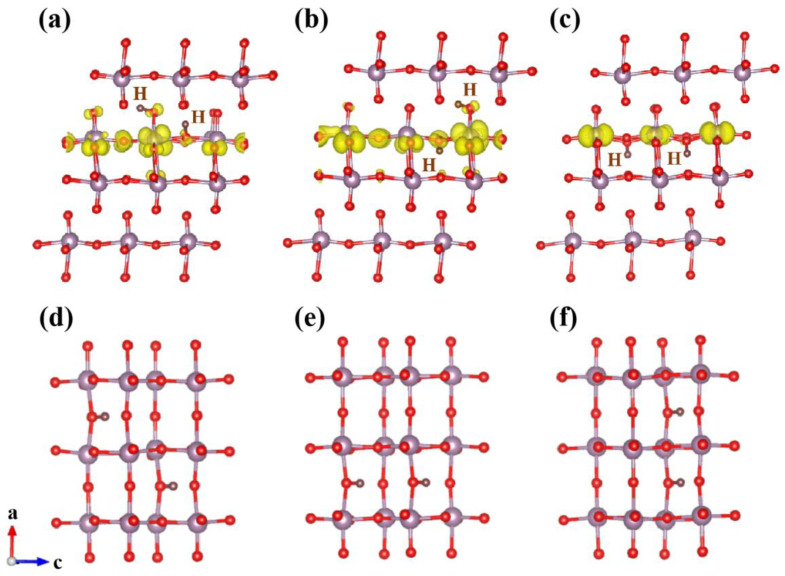
(**a**) Partial charge density distribution of gap state in AA site. (**b**) Partial charge density distribution of gap state in AB site. (**c**) Partial charge density distribution of gap state in BB site. (**d**) BB state [101] direction. (**e**) BB state [001] direction. (**f**) BB state [100] direction.

As shown in Figure 4b,c, when H is doped in AA or AB mode, Mo-4d is strongly coupled with O-2p orbit due to the bonding of H-O_t_ and H-O_a_. As a result, the orbital electrons are delocalized and an obvious trap state is formed in the middle of the forbidden band. When H is doped in BB mode, due to the bonding of H-O_a_, Mo-4d and O-2p, it only produces weak coupling and the trap state is near the bottom of the conduction band, which is mainly contributed by the Mo-4d state (as shown in Figure 4d). Furthermore, the partial charge distribution in Figure 5 is consistent with the conclusion above. In BB doping mode, H atoms lose electrons and become protons (Figure 5c). The charge of hydrogen atoms is mainly transferred to O_a_ atoms in the same layer. The charge transfer of O_a_ required bonding with Mo is reduced, resulting in an impure state contributed primarily by the Mo-4d orbital. In AA and AB doping modes, the charge of hydrogen atoms is transferred to nearby Mo and O atoms at the same time, and a large amount of charge is localized, resulting in strong Mo-4d and O-2p orbital coupling. The deep level defects are formed eventually, as in the previous calculation results for density of states (see Figure S1 for band structure). The phenomenon of electron transfer from H atoms to MoO_3_ can also be observed by experimental Raman spectroscopy. T. Hirata et al. reported that the Raman mode of MoO_3_ would decrease in intensity with the increase in H+ concentration implanted, which indicates the electron transfer from hydrogen to MoO_3_ [34]. In addition, from the effect of band gap modulation it is clear that H atom doping at higher concentration can tune the band gap in a wider range. When there are H atoms in the interlayer, H is bonded with O_t_ and O_a_ in the same octahedron, which also improves the conductivity of α-MoO_3_ and optimizes its optoelectronic properties. This phenomenon is mainly due to the introduction of deeper impurity states between bands, which improves the transition probability of carriers. In the case where H atoms only exist in the intralayer, they tend to bond with O_a_ and the total system energy of H doped α-MoO_3_ is the lowest, showing a similar regularity to that observed in low concentration H-doping calculations. Our work also calculated the 25% doping concentration, which shows a similar behavior with the doping concentration of 8%. The total energy of the doped system shows that the most stable doping situation is still BB site, when all the H atoms are doped in intralayer (see Table S3 for detailed data). The trap state is further extended and shows the strong coupling between Mo-4d orbit and O-2p orbit (see Figure S2 for DOS at 25% concentration). As shown in Figure 5d–f, we also found that both H atoms tend to bond with asymmetric oxygen at site BB, and the energy of H atoms arranged along [001] or [101] direction is 0.5 eV lower than that along [100] direction (see Table S4 for detailed data).

**Figure 6:**
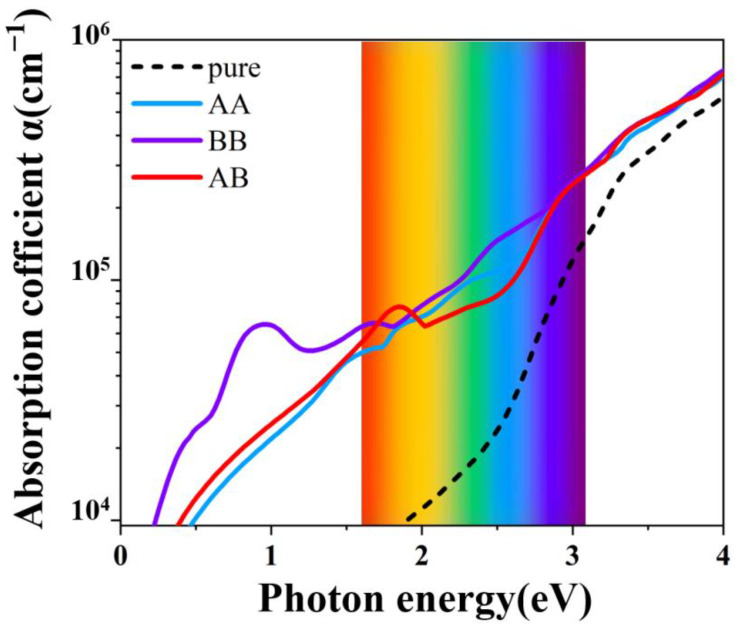
AA, AB, BC, and pure α-MoO_3_ light absorption.

Finally, the optical absorption spectra for different doping situations and the different modulation on the optical properties of the systems were investigated. We calculated the optical absorption coefficients of three different doped systems (AA, AB, BB). As shown in Figure 6, compared with the α-MoO_3_ system before doping, the light absorption coefficient of the doped system was significantly improved in the visible and infrared regions. These findings support the results by M. H. Yaacob et al., which list the absorbance versus optical wavelength of the Pd/MoO_3_ film and show that the magnitude of the absorbance was increased significantly when the film was exposed to 1% H_2_ in synthetic air [35] (see Figure S3 for light absorption at different doping concentration). Particularly, in the most stable BB doped system, its optical absorption property behaves best in the long wavelength visible region and the infrared region.

## 4. Conclusions

We used first-principles calculation to study the physical mechanism of changing optoelectronic properties in H doped α-MoO_3_. At low concentration (4%) doping, hydrogen atoms tend to be distributed in the intralayer, and H-O bonding causes local lattice distortion of α-MoO_3_, which leads to an impurity gap state near the minimum of the conduction band. At high concentration (8%) doping, the impurity gap state is expanded, which reduces the band gap to an extreme. At the concentration of 8%, we set three different doping positions: AA, AB, and BB. The AA and AB doping states lead to strong coupling between Mo-4d and O-2p orbits, and finally produce impurity extended states located deep in the forbidden band. As a result, the high concentration dopant will tune the band gap and optoelectronic properties of the material in a wider range. Interestingly, when H atoms only exist in the intralayer (BB state), the doped MoO_3_ system is the most stable one and the impurity state is mainly contributed by Mo-4d orbital near the conduction band. Hydrogen atoms in the BB doping state were found to have a tendency arranging towards [101] or [001] and create a remarkable effect on enhancing light absorption.

## Data Availability

Not applicable.

## Supplementary Materials

The following supporting information can be downloaded at: https://www.mdpi.com/article/10.3390/ma15093378/s1, Figure S1: Band structure of hydrogen doped at AA, AB, BB position at 8% concentration. (a) Band structure of AA position. (b) Band structure of AB position. (c) Band structure of BB position. Figure S2: DOS of hydrogen doped at AA, AB, BB position at 25% concentration. (a) Density of states of AA site. (b) Density of states of AB site. (c) Density of states of BB site. Figure S3: light absorption of different doping concentration. Table S1: The total energy of systems in A1, A2, A3, B1, B2 doping sites. Table S2: The total energy of systems with different 8% concentration doping site. Table S3: The total energy of systems with different 25% concentration doping site. Table S4: The total energy of BB systems in different crystal orientation. Table S5: Interlayer spacing of different doping concentration.
