# Supplementary material for: Optoelectronic Properties of α-MoO3 Tuned by H Dopant in Different Concentration
Source: Materials (Basel). 2022 May 8;15(9):3378. doi: 10.3390/ma15093378 (PMC9101102; doi:10.3390/ma15093378)
Supplement: Supplementary file 1 [file materials-15-03378-s001.zip › materials-1668063-supplementary.pdf]

Supporting Information

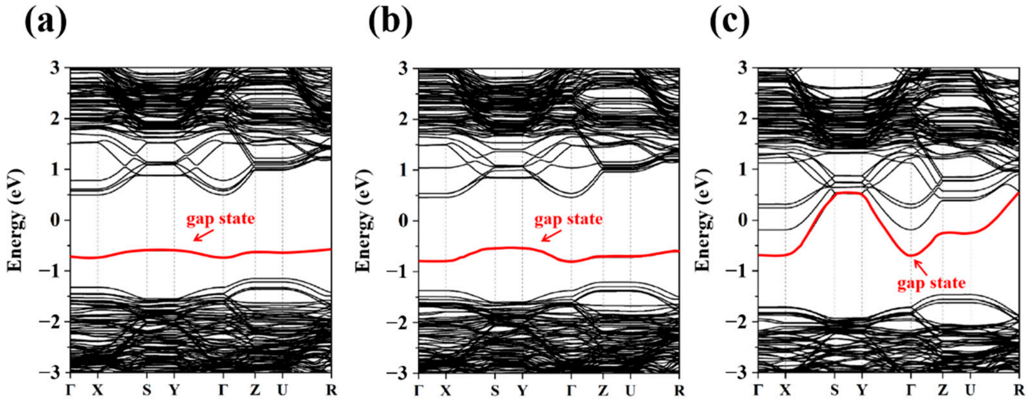

Figure S1. Band structure of hydrogen doped at AA, AB, BB position at 8% concentration. (**a**) Band structure of AA position. (**b**) Band structure of AB position. (**c**) Band structure of BB position.

The positions of the gap states caused by AA and AB doping site are basically the same, and move obviously to the maximum of the valence band compare to the BB state.

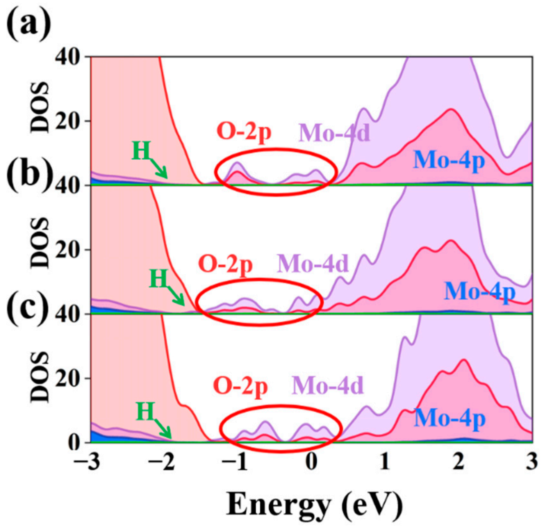

Figure S2. DOS of hydrogen doped at AA, AB, BB position at 25% concentration. (**a**) Density of states of AA site. (**b**) Density of states of AB site. (**c**) Density of states of BB site.

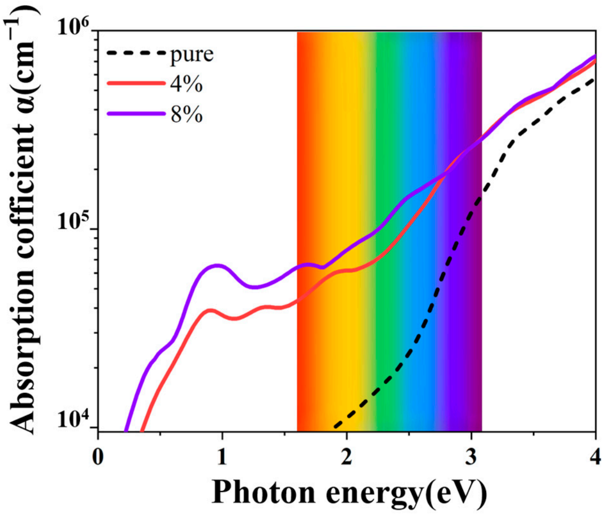

Figure S3. light absorption of different doping concentration.

Table S1. The total energy of systems in A_1， A_2， A_3， B_1， B_2 doping sites.

Interlayer	Total Energy/eV
A_1	-613.400
	-613.401
	-613.400
A_2	-613.690
	-613.581
A_3	-613.235
Intralayer	Total Energy/eV
B_2	-613.868
B_3	-613.194

Table S2. The total energy of systems with different 8% concentration doping site.

Different doping site	Different doping site	Total Energy/eV
AA	O_tO_t	-617.110
	O_tO_a	-617.620
	O_aO_a	-617.376
AB	O_aO_a	-617.597
	O_tO_a	-617.828
BB	O_aO_a	-618.060

Table S3. The total energy of systems with different 25% concentration doping site.

Different doping site	Total Energy/eV
	-631.503
AB	-631.822
BB	-632.169

Table S4. The total energy of BB systems in different crystal orientation.

Crystal orientation	Total Energy/eV
[101]	-618.06
[001]	-618.06
[100]	-617.52

Table S5. Interlayer spacing of different doping concentration.

Different doping concentration	Interlayer spacing/Å
4%	5.012
8%	5.014
